# Analysis of long-term strategies of riparian countries in transboundary river basins

**DOI:** 10.1038/s41598-021-99655-5

**Published:** 2021-10-12

**Authors:** Fahimeh Mirzaei-Nodoushan, Omid Bozorg-Haddad, Vijay P. Singh, Hugo A. Loáiciga

**Affiliations:** 1 Department of Irrigation & Reclamation Engineering, Faculty of Agricultural Engineering & Technology, College of Agriculture & Natural Resources, University of Tehran, Karaj, Tehran, Iran; 2 Caroline & William N. Lehrer Distinguished Chair in Water Engineering, Department of Biological and Agricultural Engineering & Zachry Department of Civil & Environmental Engineering, Texas A&M University, 321 Scoates Hall, 2117 TAMU, College Station, TX 77843-2117 USA; 3 Department of Geography, University of California, Santa Barbara, CA 93106 USA

**Keywords:** Climate sciences, Ecology, Environmental sciences, Environmental social sciences, Hydrology, Natural hazards, Energy science and technology, Engineering, Mathematics and computing

## Abstract

Transboundary river basins give rise to complex water-sharing decision making that can be analyzed as a game in the sense of dynamic game theory, as done in this work. The sharing of transboundary water resources depends on the long-term shifting interactions between upstream and downstream countries, which has received limited research attention in the past. The water-sharing strategy of a riparian country depends on the strategies of other countries over time. This paper presents an evolutionary game method to analyze the long-term water-sharing strategies of countries encompassing transboundary river basins over time. The method analyzes the evolutionary strategies of riparian countries and investigates evolutionary stable strategies (ESSs) considering the payoff matrix. The evolutionary game method is applied to a river basin shared by three countries assuming two types of benefits and one type of cost to countries as decision variables of a game that reflects water use, economic and political gains, and socio-economic losses of countries. Numerical examples illustrate the strategies resulting from the evolutionary game processes and the role of several parameters on the interaction between riparian countries. The countries’ strategies are analyzed for several levels of benefits and costs, and the convergence of the strategies to a stable point is assessed. Results demonstrate the role that the upstream country’s potential benefits and the cost of conflict (i.e., non-cooperation) to other countries has on reaching a stable point in the game. This work’s results show the potential benefit to the upstream country under cooperative strategy must exceed its benefits from water use under non-cooperative strategy to gain the full stable cooperation of downstream countries. This work provides a method to resolve water-sharing strategies by countries sharing transboundary river basins and to evaluate the implications of cooperation or non-cooperation.

## Introduction

Freshwater is a vital resource for humans and the environment. Growing population, rapid urban, agricultural, and industrial development, and climate change lead to increasing water demands that make water resources more vulnerable. Water sharing between countries with different approaches to water use and policy making may cause conflicts involving shared water resources (rivers, lakes, aquifers). Management of a transboundary rivers, in particular, involves engineering, environmental, legal, social, economic, and political factors that may not be easily resolved. Yet, there may be substantial benefits from water sharing by riparian countries that are superior to the results of non-cooperation. Therefore, strategic decision making concerning the sharing of water resources may be imperative to gain the most benefits for all the parties involved.

Choosing strategic decisions by riparian countries depends on the benefits and costs of countries’ interactions. The transboundary water sharing becomes more complex as the number of countries sharing the basin increases. Each country tends to use as much water as possible from the shared water, yet, all countries must consider their combined impacts seeking to ensure equitable sharing of the benefits and costs of water use, and avoid the tragedy of the commons^1^. It is imperative when resolving transboundary water conflicts to study the strategies of the involved countries to attain the best payoff for all riparian countries.

Studies on conflict resolution concerning transboundary river basins have focused on river water allocation by means of optimization models and game theory. The common purpose of these studies is allocating water and benefits stemming from cooperation, or the lack of it, between all riparian countries. Mahjouri and Ardestani^2^ applied and compared two cooperative and non-cooperative methods for large-scale water allocation of a river in Southern Iran. They depicted the importance of cooperation in water use considering water quantity and quality issues. Kazemi et al.^3^ presented a multi-objective optimization model for water allocation in the Sefidrud basin located in Iran. Under cooperation water was apportioned among provinces so as to maximize revenue and minimize the Gini index, which led to equity in water allocation. Liu et al.^4^ applied a fuzzy coalition game model for water allocation in the Lancang-Mekong River in East Asia and Southeast Asia. The latter authors implemented the Shapley value to distribute the water utility among countries relying on cooperative strategies. The above studies and others have considered the cooperative and non-cooperative strategies of water users to distribute water among stakeholders. One of the key assumptions made by most studies dealing with game theory in transboundary river basins is that countries located in a transboundary river basin act rationally and their decisions are logical. In traditional game theory the uncertainty of decisions is neglected and the strategies of countries located in a transboundary river basin are constant over time. An assessment of the probable cooperation between riparian countries requires factoring in the influence of uncertainty on their strategies.

An evolutionary game is a mathematical model of strategic interactions between players that may evolve over time^5^. The stable strategies of game players in an evolutionary game are called evolutionary stable strategies (ESSs). Evolutionary game theory provides the tools to explore the evolution of players’ strategies and to analyze the effectiveness of payoff parameters. The initial strategies of players may change throughout the game according to the uncertainty of players’ strategies. The long-term strategies of players reflect the variability of the strategies in the evolutionary game theory over time. Evolutionary game theory has been applied in various fields, such as urban institutions^6–8^, internet networks^9^^,^^10^, and water resources and the environment^11–16^. These studies focused on players’ behaviors and their dynamic strategies, and assessed the sensitivity of strategies to problem parameters. Few studies have applied evolutionary game theory in the field of transboundary water management. Li^11^ analyzed the strategic choices of water users by evolutionary game theory with a focus on water quality. All countries of the basin had the goal of maximizing economic and environmental benefits. It was assumed that water pollution is taxed by government regulations. Tian et al.^14^ applied evolutionary game theory to assess the transboundary water conflicts between two countries that have water-trading rights. Their results demonstrated the application of water-rights trading and the water market to solving water conflicts. Yuan et al.^15^ combined evolutionary game theory and a system dynamics model to find equilibrium outcomes of strategic scenarios of two countries located in a transboundary river basin.

The literature on transboundary water management dealing with evolutionary game theory highlights countries’ dual interactions that did not extent beyond two countries. As the number of transboundary countries grows so does the complexity of the payoff matrix. Also, the political benefits and social, economic, and environmental costs have not been considered or not explicitly accounted for in previous studies. A review of the pertinent literature on transboundary river sharing reveals a neglect of impact analysis of changing payoff parameters on evolutionary stable strategies.

This work applies the concept of evolutionary game theory to understand the evolving decision-making concerning water sharing by the riparian countries encompassing a transboundary river basin. Specifically, this study tackles a tripartite transboundary water-sharing problem with evolutionary game theory. There are one upstream and two downstream countries. All the combinations of the countries’ strategies and their payoffs under the strategies are defined, and the payoff matrix of a tripartite game is developed. Two types of benefits are introduced in the game model according to the characteristics of transboundary water problems, which are water benefits and potential benefits. A cost parameter is added to the payoff matrix that represents the cost levied on a country with non-cooperative strategies. This paper demonstrates how changing the parameters of the transboundary water problem would change the equilibrium water-sharing strategies of countries. This work calculates the countries’ probabilities of cooperation or non-cooperation according to the benefits and costs of the probable strategies, and illustrates the benefit and cost parameters effectiveness in determining the water-sharing strategies of the transboundary countries.

The paper is organized as follows. “Evolutionary game theory” section describes evolutionary game theory and compares it with traditional game theory. “Evolutionary game model for transboundary river basins” section describes the evolutionary game model for transboundary river basins, presents the basic assumptions, parameters and variables, and constructs the model for tri-country water sharing. “Numerical examples” section presents numerical examples quantifying the effect of the parameters on the evolutionary strategies. The paper’s conclusions are found in “Conclusion” section.

## Evolutionary game theory

Game theory has been widely used to model social interactive situations^17^. Traditional game theory refers to a game in which players of a strategic game choose their decisions at the same time or in which players choose decisions at different times, and no player has any information about other players’ choices^18^. Evolutionary game theory, on the other hand, is based on a game in which each player chooses his decisions over time and knows other players’ previous decisions.

A significant difference between game theory and evolutionary game theory is the nature of the actors of a game. In traditional game theory the game actors are players who have fixed psyches during the game. The central actor of evolutionary game theory, on the other hand, is a replicator who makes relevantly accurate copies of itself. The replicator can be a gene, a strategy, a technique, an idea, etc.^19^. A replicator system is a set of replicators that changes frequently, and in which successful replicators reproduce more quickly than less successful ones. Central to evolutionary game theory is the probabilistic nature of decision making.

The traditional game theory is a static game and assumes that the players decide rationally. Based on the assumption that all players of the game act rationally means that all players would use dominant strategies or strategies leading to Nash equilibrium^17^. In contrast, evolutionary game theory overcomes the rational limitation of players in a game and considers the dynamic process of the game^20–23^. Therefore, the players’ strategies are not constant and they may change from time to time over repeated iterations of the game. In evolutionary game theory, players’ strategies lead to evolutionary equilibrium. Evolutionary stable strategies (ESSs) were introduced by Maynard Smith and Price^24^ as a refinement of the Nash equilibrium to deal with evolutionary games. An ESS is a strategy that cannot be overcome or changed by other strategies in a repeated game.

Evolutionary game theory is herein applied to transboundary river basins in which the strategies of riparian countries change over time.

## Evolutionary game model for transboundary river basins

Assume *n* countries ($n\ge 2$) are located in a transboundary river basin and they are the players of an evolutionary game in which the countries’ strategies concerning water sharing in the basin evolve over time. Each country can choose between a cooperative strategy or a non-cooperative strategy. The game’s interactions and players’ payoffs vary with the number *n* and the location of the countries within the river basin, specifically, in relation to whether they are upstream-located or downstream-located countries within the river basin. The probability of country *i* choosing a cooperative strategy is herein denoted by ${x}_{1}^{(i)}$, $i=1, 2, \dots , n$, and there are ${2}^{n}$ payoff sets for all the combinations of the countries’ strategies. This paper assesses the interactions between three countries sharing a transboundary river basin.

### Problem description

Let 1, 2, and 3 denote three countries sharing a transboundary river basin. Country 1 is upstream and countries 2 and 3 are located downstream. Country 1 can use maximum amount of the water of the river and choose not to share it with the downstream countries. This strategy, however, may trigger conflict with the two other countries of political, social, economic, security, and environmental natures. Instead, Country 1 can release excess water to be shared by Countries 2 and 3. Countries 2 and 3 are inclined to cooperate with Country 1 unless other benefits emerge by being non-cooperative with Country 1.

There are two types of benefits and one type of cost in the payoff matrix of the assumed problem that are economic in nature. The first is a water benefit earned by a country from receiving the water from the transboundary river. The set of benefits related to water use includes economic benefits earned from agricultural, urban, and industrial development benefits. It should be noted that the water benefit for Country 1 means the economic benefit of consuming more water than its water right from the river. So, water benefits of Country 2 and 3 are the economic benefit of consuming excess water of upstream which is released by Country 1.

**Figure 1:**
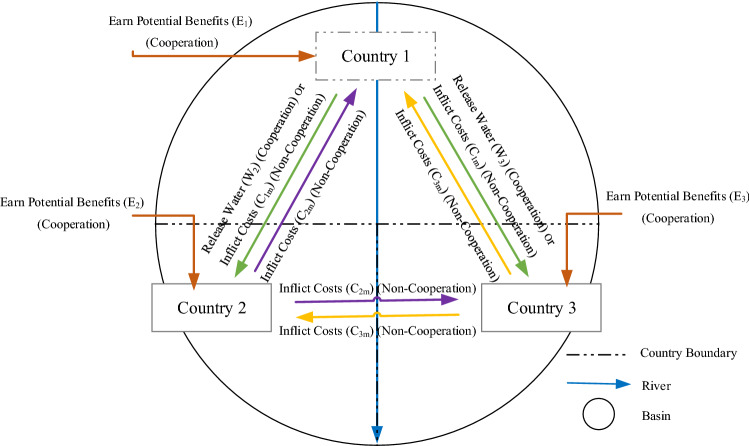
Schematic of the transboundary river and riparian countries with their shifting interactions.

The second is a potential benefit earned from the cooperative strategy of a country. Cooperation benefits stem from sustainability conditions like social interests, environmental benefits and political conjunctures such as international alliances and harmony from amicable interactions with neighboring countries. The parameters *F* and *E* (water benefit and potential benefit, respectively) encompass a number of benefit parameters; nevertheless, parameters were simplified to two benefit parameters to simplify the complexity of the water-sharing problem. Costs forced on other countries from non-cooperation by a country involves commercial, security, political, diplomatic, military, and environmental costs. Figure 1 displays the locations of three countries and their shifting interactions in a transboundary river basin.

### Basic assumptions

The evolutionary game model of interactions between riparian countries in the transboundary river basin rests on the following assumptions:

#### **Assumption 1**

There are three countries (i.e., players) in the game of transboundary water sharing, each seeking to maximize its payoff from the game.

#### **Assumption 2**

Country 1 has two possible strategies. One is for Country 1 to release a specified amount of water to the downstream countries (this would be Country 1’s cooperative strategy). The cooperative strategy by Country 1 would produce benefits *F*_2_ and *F*_3_ to Countries 2 and 3, respectively. By being cooperative Country 1 would attain a benefit *E*_1_ called the potential benefit from cooperative responses from the downstream countries. The other strategy is for Country 1 to deny water to the downstream countries (this would be Country 1’s non-cooperative strategy), in which case Country 1 would earn the water benefit *F*_1_ from using water that would otherwise be released, but would forego the potential benefit *E*_1_. Moreover, by pursuing a non-cooperative strategy Country 1 would inflict a cost *C*_1*m*_ to the downstream countries.

#### **Assumption 3**

There are two possible strategies for Country 2. One is for Country 2 to accept the behavior of Country 1 (this would be Country 2’s cooperative strategy), which would cause earning a potential benefit *E*_2_ to Country 2. Recall that if Country 2 acquiesces to Country 1’s cooperative behavior it would receive a benefit *F*_2_. Or, Country 2 may disagree with Country 1 (this would be Country 2’s non-cooperative strategy), in which case, Country 2 would lose benefit *E*_2_*,* and it would inflict a cost *C*_2*m*_ to the other countries.

#### **Assumption 4**

Similar to Country 2, Country 3 has two possible strategies. One is for Country 3 to agree Country 1’s behavior (this would be Country 3’s cooperative strategy) attaining a potential benefit *E*_3_. Recall that if Country 3 agrees with Country 1’s cooperative behavior it would gain a benefit *F*_3_. Another strategy for Country 3 is to oppose Country 1 (this would be Country 3’s non-cooperative strategy) missing the benefit *E*_3_ and forcing a cost *C*_3*m*_ to the other countries.

Table 1 defines the benefits and costs that enter in the transboundary water-sharing game described in this work. The payoff to country $i=\mathrm{1,2},3$ depends on its own strategy and on the strategies of the other countries, and each country may choose to be cooperative or non-cooperative. The strategies of country $i$ are denoted by 1 (cooperation) and 2 (non-cooperation). The probabilities of country $i$’s strategies are denoted by ${x}_{1}^{(i)}$ and by ${x}_{2}^{(i)}$, in which the former represents cooperation and the latter represents non-cooperation. Clearly, ${x}_{1}^{(i)}$+ ${x}_{2}^{(i)}$ = 1. The payoff to country $i=1, 2, 3$ when the strategies of Countries 1, 2, 3 are $j, k,l$, respectively, where $j, k,l$ may take the value 1 (cooperation) or 2 (non-cooperation) is denoted by ${U}_{jkl}^{\left(i\right)}$. Thus, for instance, the payoff to country $i=2$ is represented by ${U}_{212}^{(2)}$ when Countries 1 and 3 are non-cooperative and Country 2’s strategy is cooperative. Evidently, there are 2^3^ payoffs to each country given there are three countries involved and each can be cooperative or non-cooperative. Table 2 shows the symbols for the payoffs that accrue to each country under the probable strategies.

**Table 1:** Benefits and costs.

Player	Parameter	Description
Country 1	${x}_{1}^{(1)}$*,* ${x}_{2}^{(1)}$	Probabilities of Country 1 cooperating or not cooperating, respectively
	*F*_1_	Water benefit for Country 1
	*E*_1_	Potential benefit due to cooperation of Country 1
	*C*_11_	Cost inflicted on Country 2 due to non-cooperation of Country 1 when Country 2 and 3 are cooperative
	*C*_12_	Cost inflicted on Country 3 due to non-cooperation of Country 1 when Country 2 and 3 are cooperative
	*C*_13_	Cost inflicted on Country 2 due to non-cooperation of Country 1 when Country 2 is cooperative and Country 3 is not cooperative
	*C*_14_	Cost inflicted on Country 3 due to non-cooperation of Country 1 when Country 2 is cooperative and Country 3 is not cooperative
	*C*_15_	Cost inflicted on Country 2 due to non-cooperation of Country 1 when Country 2 is not cooperative and Country 3 is cooperative
	*C*_16_	Cost inflicted on Country 3 due to non-cooperation of Country 1 when Country 2 is not cooperative and Country 3 is cooperative
	*C*_17_	Cost inflicted on Country 2 due to non-cooperation of Country 1 when Country 2 and 3 are not cooperative
	*C*_18_	Cost inflicted on Country 3 due to non-cooperation of Country 1 when Country 2 and 3 are not cooperative
Country 2	${x}_{1}^{(2)}$*,* ${x}_{2}^{(2)}$	Probabilities of Country 2 cooperating or not cooperating, respectively
	*F*_2_	Water benefit for Country 2
	*E*_2_	Potential benefit due to cooperation of Country 2
	*C*_21_	Cost inflicted on Country 1 due to non-cooperation of Country 2 when Country 1 and 3 are cooperative
	*C*_22_	Cost inflicted on Country 3 due to non-cooperation of Country 2 when Country 1 and 3 are cooperative
	*C*_23_	Cost inflicted on Country 1 due to non-cooperation of Country 2 when Country 1 is cooperative and Country 3 is not cooperative
	*C*_24_	Cost inflicted on Country 3 due to non-cooperation of Country 2 when Country 1 is cooperative and Country 3 is not cooperative
	*C*_25_	Cost inflicted on Country 1 due to non-cooperation of Country 2 when Country 1 is not cooperative and Country 3 is cooperative
	*C*_26_	Cost inflicted on Country 3 due to non-cooperation of Country 2 when Country 1 is not cooperative and Country 3 is cooperative
	*C*_27_	Cost inflicted on Country 1 due to non-cooperation of Country 2 when Country 1 and 3 are not cooperative
	*C*_28_	Cost inflicted on Country 3 due to non-cooperation of Country 2 when Country 1 and 3 are not cooperative
Country 3	${x}_{1}^{(3)}$*,* ${x}_{2}^{(3)}$	Probabilities of Country 3 cooperating or not cooperating, respectively
	*F*_3_	Water benefit for Country 3
	*E*_3_	Potential benefit due to cooperation of Country 3
	*C*_31_	Cost inflicted on Country 1 due to non-cooperation of Country 3 when Country 1 and 2 are cooperative
	*C*_32_	Cost inflicted on Country 2 due to non-cooperation of Country 3 when Country 1 and 2 are cooperative
	*C*_33_	Cost inflicted on Country 1 due to non-cooperation of Country 3 when Country 1 is cooperating and Country 2 is not cooperative
	*C*_34_	Cost inflicted on Country 2 due to non-cooperation of Country 3 when Country 1 is cooperating and Country 2 is not cooperative
	*C*_35_	Cost inflicted on Country 1 due to non-cooperation of Country 3 when Country 1 is not cooperative and Country 2 is cooperative
	*C*_36_	Cost inflicted on Country 2 due to non-cooperation of Country 3 when Country 1 is not cooperative and Country 2 is cooperative
	*C*_37_	Cost inflicted on Country 1 due to non-cooperation of Country 3 when Country 1 and 2 are not cooperative
	*C*_38_	Cost inflicted on Country 2 due to non-cooperation of Country 3 when Country 1 and 2 are not cooperative

**Table 2:** Payoff matrix under cooperation or non-cooperation.

Country 1	Country 2	Country 3	
		Cooperation (${x}_{1}^{(3)}$)	Non-cooperation (${x}_{2}^{(3)}$)
Cooperation (${x}_{1}^{(1)})$	Cooperation (${x}_{1}^{(2)}$)	(${U}_{111}^{\left(1\right)},{U}_{111}^{\left(2\right)},{U}_{111}^{\left(3\right)})$	(${U}_{112}^{(1)},{U}_{112}^{(2)},{U}_{112}^{(3)})$
	Non-cooperation (${x}_{2}^{(2)}$)	(${U}_{121}^{(1)},{U}_{121}^{(2)},{U}_{121}^{(3)})$	(${U}_{122}^{(1)},{U}_{122}^{(2)},{U}_{122}^{(3)})$
Non-cooperation (${x}_{2}^{(1)}$)	Cooperation (${x}_{1}^{(2)})$	(${U}_{211}^{(1)},{U}_{211}^{(2)},{U}_{211}^{(3)})$	(${U}_{212}^{(1)},{U}_{212}^{(2)},{U}_{212}^{(3)})$
	Non-cooperation (${x}_{2}^{(2)}$)	(${U}_{221}^{\left(1\right)},{U}_{221}^{\left(2\right)},{U}_{221}^{\left(3\right)})$	(${U}_{222}^{(1)},{U}_{222}^{(2)},{U}_{222}^{(3)})$

${x}_{1}^{(i)}$ and ${x}_{2}^{(i)}$, represents the probabilities of country $i$ (= 1, 2, 3) acting cooperatively or non-cooperatively, respectively. Notice that ${x}_{1}^{(i)}+{x}_{2}^{(i)}$ = 1.

### Formulation of the transboundary water-sharing strategies as an evolutionary game

The expected payoff to country $i$ is expressed by the following equation:

1 $${U}^{(i)}=\sum\limits_{j = 1}^2 {\sum\limits_{k = 1}^2 {\sum\limits_{l = 1}^2} } {x}_{j}^{(1)}{x}_{k}^{(2)}{x}_{l}^{(3)} {U}_{jkl}^{(i)} \quad i=1, 2, 3$$

The following describe the expected payoffs of Country 1 when it acts cooperatively (${U}_{1}^{(1)})$ or non-cooperatively (${U}_{2}^{(1)}$):

2 $${U}_{1}^{(1)}={x}_{1}^{(2)}{x}_{1}^{(3)}{U}_{111}^{\left(1\right)}+{x}_{1}^{(2)}{x}_{2}^{(3)}{U}_{112}^{\left(1\right)}+{x}_{2}^{(2)}{x}_{1}^{(3)}{U}_{121}^{(1)}+{x}_{2}^{(2)}{x}_{2}^{(3)}{U}_{122}^{(1)}$$

3 $${U}_{2}^{(1)}={x}_{1}^{(2)}{x}_{1}^{(3)}{U}_{211}^{\left(1\right)}+{x}_{1}^{(2)}{x}_{2}^{(3)}{U}_{212}^{\left(1\right)}+{x}_{2}^{(2)}{x}_{1}^{(3)}{U}_{221}^{\left(1\right)}+{x}_{2}^{(2)}{x}_{2}^{(3)}{U}_{222}^{\left(1\right)}$$

Therefore, the expected payoff of Country 1 is ${U}^{(1)}$ which is equal to:

4 $${U}^{(1)}={x}_{1}^{(1)}{U}_{1}^{(1)}+{x}_{2}^{(1)}{U}_{2}^{(1)}= \sum\limits_{j = 1}^2 {\sum\limits_{k = 1}^2 {\sum\limits_{l = 1}^2 } }{x}_{j}^{(1)}{x}_{k}^{(2)}{x}_{l}^{(3)} {U}_{jkl}^{(1)}$$

The expected payoffs of Countries 2 and 3 can be similarly obtained as done for Country 1. The cooperative and non-cooperative expected payoffs of all countries can be expressed in terms of the payoffs listed in Table 1. The results are found in Appendix A.

### Replication dynamics equations

The replication dynamics equations describe the time change of the probabilities of a player’s strategies. The replication dynamics equation of Countries $i$ is denoted by ${G}^{(i)}\left({x}_{1}^{(i)}\right)$ which is as follow^22^:

5 $${G}^{(i)}\left({x}_{1}^{(i)}\right)=\frac{d{x}_{1}^{(i)}}{dt}={x}_{1}^{(i)}\left({U}_{1}^{(i)}-{U}^{(i)}\right)$$

The replication dynamics equations of Countries 1, 2 and 3 are presented in Appendix B according to the benefits and costs showed in Table 1.

### Stability analysis of a country’s strategies

Under the assumption of bounded rationality each country does not know which strategies may lead to the optimal solution in the game. Therefore, the countries’ strategies change over time until a stable (i.e., time-independent) solution named evolutionary stable strategy (ESS) is attained. The evolutionary stable theorem for replication dynamics equation states that a stable probability of cooperation ${x}_{1}^{(i)}$ for country $i$ occurs if the following conditions hold^25^: (1) ${G}^{(i)}\left({x}_{1}^{(i)}\right)=0$, and (2) $d{G}^{(i)}\left({x}_{1}^{(i)}\right)/d{x}_{1}^{(i)}<0$ when evaluated at ${x}_{1}^{(i)}$, that is, when ${G}^{(i){^{\prime}}}\left({x}_{1}^{(i)}\right)<0$. The probability of cooperation ${x}_{1}^{(i)}$ represents an ESS point if the former two conditions apply. Evidently, a stable probability of non-cooperation equals $1-{x}_{1}^{(i)}$ when ${x}_{1}^{(i)}$ exists. The method to obtain the stability probabilities, when they exist, is herein described in full for Country 1. It can be concluded from Equation (B1) of Appendix B that ${G}^{\left(1\right)}\left({x}_{1}^{\left(1\right)}\right)={x}_{1}^{\left(1\right)}{x}_{2}^{\left(1\right)}{A}_{0}\left({x}_{1}^{\left(2\right)},{x}_{1}^{\left(3\right)}\right)=0$ if any of the following occurs: (i) ${x}_{1}^{(1)}=0$, (ii) ${x}_{1}^{(1)}=1$, (iii) ${A}_{0}\left({x}_{1}^{(2)},{x}_{1}^{(3)}\right)=0$. Condition (iii) holds if the following is true (the constants ${a}_{1}$, ${a}_{2}$, ${a}_{3}$, ${a}_{4}$ are defined in Equation (B1) of Appendix B):

6 $${x}_{1}^{(2)}=-\frac{{a}_{3 }{x}_{1}^{(3)}+{a}_{4}}{{a}_{1 }{x}_{1}^{(3)}+{a}_{2}}$$

Furthermore, ${G}^{(1){^{\prime}}}=d{G}^{(1)}/d{x}_{1}^{(1)}=\left(1-2{x}_{1}^{(1)}\right){A}_{0}\left({x}_{1}^{(2)},{x}_{1}^{(3)}\right)$. Therefore, the ESSs of Country 1 are evaluated as follows:

1. If ${x}_{1}^{(2)}=-\frac{{a}_{3}{x}_{1}^{(3)}+{a}_{4}}{{a}_{1}{x}_{1}^{(3)}+{a}_{2}}$, then ${A}_{0}=0$, ${G}^{\left(1\right)}\left({x}_{1}^{\left(1\right)}\right)=0$, and ${G}^{{\left(1\right)}^{{\prime}}}\left({x}_{1}^{\left(1\right)}\right)=0$, which means Country 1’ probabilities remain invariant over time;
2. If $0<{x}_{1}^{(2)}<-\frac{{a}_{3}{x}_{1}^{(3)}+{a}_{4}}{{a}_{1}{x}_{1}^{(3)}+{a}_{2}}$, then ${A}_{0}<0$ and ${G}^{\left(1\right){^{\prime}}}\left({x}_{1}^{\left(1\right)}=0\right)<0$. So ${x}_{1}^{\left(1\right)}=0$ is the ESS in which Country 1’s ESS is non-cooperative;
3. If $-\frac{{a}_{3}{x}_{1}^{\left(3\right)}+{a}_{4}}{{a}_{1}{x}_{1}^{\left(3\right)}+{a}_{2}} <{x}_{1}^{\left(2\right)}<1$, then ${A}_{0}>0$ and ${G}^{\left(1\right){^{\prime}}}\left({x}_{1}^{\left(1\right)}=1\right)<0$. Thus ${x}_{1}^{\left(1\right)}=1$ is the ESS in which Country 1’s ESS is cooperative.

The procedure for determining Country 1’s ESS was applied to Countries 2 and 3 to determine their ESSs. The results are as follows:

Country 2’s ESSs (the constants ${b}_{1}$, ${b}_{2}$, ${b}_{3}$, ${b}_{4}$ are defined in Equation (B6) Appendix B):

1. If ${x}_{1}^{(1)}=-\frac{{b}_{3}{x}_{1}^{(3)}+{b}_{4}}{{b}_{1}{x}_{1}^{(3)}+{b}_{2}}$, then ${B}_{0}=0$, ${G}^{\left(2\right)}\left({x}_{1}^{\left(2\right)}\right)=0$, and ${G}^{{\left(2\right)}^{{\prime}}}\left({x}_{1}^{\left(2\right)}\right)=0$, which means Country 2’ probabilities remain invariant over time;
2. If $0<{x}_{1}^{(1)}<-\frac{{b}_{3}{x}_{1}^{(3)}+{b}_{4}}{{b}_{1}{x}_{1}^{(3)}+{b}_{2}}$, then ${B}_{0}<0$ and ${G}^{\left(2\right){^{\prime}}}\left({x}_{1}^{\left(2\right)}=0\right)<0$. Therefore, ${x}_{1}^{\left(2\right)}=0$ is the ESS in which Country 2’s ESS is non-cooperative;
3. If $-\frac{{b}_{3}{x}_{1}^{\left(3\right)}+{b}_{4}}{{b}_{1}{x}_{1}^{\left(3\right)}+{b}_{2}} <{x}_{1}^{\left(1\right)}<1$, then ${B}_{0}>0$ and ${G}^{\left(2\right){^{\prime}}}\left({x}_{1}^{\left(2\right)}=1\right)<0$. Thus, ${x}_{1}^{\left(2\right)}=1$ is the ESS in which Country 2’s ESS is cooperative.

Country 3’s ESSs (the constants ${c}_{1}$, ${c}_{2}$, ${c}_{3}$, ${c}_{4}$ are defined in Equation (B11) of Appendix B):

1. If ${x}_{1}^{(1)}=-\frac{{c}_{3}{x}_{1}^{(2)}+{c}_{4}}{{c}_{1}{x}_{1}^{(2)}+{c}_{2}}$, then ${C}_{0}=0$, ${G}^{\left(3\right)}\left({x}_{1}^{\left(3\right)}\right)=0$, and ${G}^{{\left(2\right)}^{{\prime}}}\left({x}_{1}^{\left(3\right)}\right)=0$, which means Country 3’ probabilities remain invariant over time;
2. If $0<{x}_{1}^{(1)}<-\frac{{c}_{3}{x}_{1}^{(2)}+{c}_{4}}{{c}_{1}{x}_{1}^{(2)}+{c}_{2}}$, then ${C}_{0}<0$ and ${G}^{\left(3\right){^{\prime}}}\left({x}_{1}^{\left(3\right)}=0\right)<0$. Thus, ${x}_{1}^{\left(3\right)}=0$ is the ESS in which Country 3’s ESS is non-cooperative;
3. If $-\frac{{c}_{3}{x}_{1}^{\left(3\right)}+{c}_{4}}{{c}_{1}{x}_{1}^{\left(3\right)}+{c}_{2}} <{x}_{1}^{\left(1\right)}<1$, then ${C}_{0}>0$ and ${G}^{\left(3\right){^{\prime}}}\left({x}_{1}^{\left(3\right)}=1\right)<0$. So, ${x}_{1}^{\left(3\right)}=1$ is the ESS in which Country 3’s ESS is cooperative.

It is evident that the evolutionary strategy of each country is dependent on the other countries’ strategies. The ESS replication dynamic equations were obtained for each country under the specified conditions.

### Stability analysis of multi-country strategies

The stable evolutionary strategies of each country were analyzed individually in the previous sections. Yet, the three countries interact with each other and their strategies may change simultaneously. Table 1 provides the possible payoffs that can arise from this paper’s game. This section evaluates the stability of the equilibrium strategies for the countries’ strategies listed in Table 2. Each country’s strategy becomes stable when the replication dynamics equations are equal to 0 (i.e., ${G}^{\left(i\right)}({x}_{1}^{\left(i\right)})$ = 0, for $i=1, 2, 3$). The solution of the set of replication dynamics equations being equal to 0 yields the equilibrium probabilities governing the countries’ ESSs (evolutionary stable strategies). Björnerstedt and Jörgen^26^ demonstrated that the stable solution to tripartite problems of the type herein considered must be a strict Nash equilibrium point, which means the probable stable solutions to the problem herein entertained are (0,0,0), (1,0,0), (0,1,0), (0,0,1), (1,1,0), (1,0,1), (0,1,1) and (1,1,1).

According to Friedman’s^5^ proposed method the Jacobian matrix of the replication dynamics system and an analysis of eigenvalues of the matrix are needed to investigate the stability of equilibrium points. The Jacobian matrix *J* for the replication dynamics system of interactions of *n* = 3 countries is as follows:

7 $$J=\left[\begin{array}{ccc}\frac{\partial {G}^{(1)}({x}_{1}^{(1)})}{\partial {x}_{1}^{(1)}}& \frac{\partial {G}^{(1)}({x}_{1}^{(1)})}{\partial {x}_{1}^{(2)}}& \frac{\partial {G}^{(1)}({x}_{1}^{(1)})}{\partial {x}_{1}^{(3)}}\\ \frac{\partial {G}^{(2)}({x}_{1}^{(2)})}{\partial {x}_{1}^{(1)}}& \frac{\partial {G}^{(2)}({x}_{1}^{(2)})}{\partial {x}_{1}^{(2)}}& \frac{\partial {G}_{2}(y)}{\partial {x}_{1}^{(3)}}\\ \frac{\partial {G}^{\left(3\right)}({x}_{1}^{\left(3\right)})}{\partial {x}_{1}^{(1)}}& \frac{\partial {G}^{\left(3\right)}({x}_{1}^{\left(3\right)})}{\partial {x}_{1}^{(2)}}& \frac{\partial {G}^{\left(3\right)}({x}_{1}^{\left(3\right)})}{\partial {x}_{1}^{(3)}}\end{array}\right]=\left[\begin{array}{ccc}{J}_{11}& {J}_{12}& {J}_{13}\\ {J}_{21}& {J}_{22}& {J}_{23}\\ {J}_{32}& {J}_{32}& {J}_{33}\end{array}\right]$$

The elements ${J}_{ij}$ of the Jacobian matrix (7) may be written in terms of the benefits and costs introduced in Table 1. The equations for the nine elements of the Jacobian matrix (7) are presented in Appendix C. The ESSs probabilities are such that all the eigenvalues of the Jacobian matrix are negative. The eigenvalues of the Jacobian matrix are obtained by solving the equation $\left|\left(J-\lambda I\right)\right|=0$ where | | denotes the determinant of a matrix, $I$ and $\lambda$ denote respectively the identity matrix and the vector of eigenvalues. The signs of eigenvalues are determined at each equilibrium point shown in Table 3. It is evident from Table 3 that there is no definitive stable solution for this problem, which is one with all certainly negative eigenvalues. Such a solution would be arrived at eventually if it existed. However, any point with uncertain stability can be an ESS point. The points (0,0,0), (1,0,0), (0,1,0), and (0,0,1) could be ESSs depending on the sign of the constants ${a}_{4}$, ${b}_{4}$, ${c}_{4}$ that appear in the replication dynamics equations. The stable point (0,0,0) occurs when no country cooperates. The constraints to reach this stable point are ${E}_{1}-{F}_{1}-{C2}_{3}-{C3}_{3}+{C2}_{7}+{C3}_{7}<0$, ${E}_{2}-{H}_{3}-{C3}_{6}+{C1}_{7}+{C3}_{8}<0$, and ${E}_{3}-{C1}_{6}-{C2}_{6}+{C1}_{8}+{C2}_{8}<0$. The constraints to achieve cooperation of the upstream country and non-cooperation of the downstream countries, i.e. (1,0,0), are ${E}_{1}-{F}_{1}-{C2}_{3}-{C3}_{3}+{C2}_{7}+C3>0$, ${E}_{2}-{C1}_{3}-{C3}_{6}+{C1}_{7}+{C3}_{8}<0$, and ${E}_{3}-{C1}_{6}-{C2}_{6}+{C1}_{8}+{C2}_{8}<0$. The stable point (0,1,0) means only the downstream Country 2 chooses to be cooperative occurs when the following constraints are met: ${E}_{1}-{F}_{1}-{C2}_{3}-{C3}_{3}+{C2}_{7}+{C3}_{7}<0$, ${E}_{2}-{H}_{3}-{C3}_{6}+{C1}_{7}+{C3}_{8}>0$, and ${E}_{3}-{C1}_{6}-{C2}_{6}+{C1}_{8}+{C2}_{8}<0$. The point (0,0,1) may occur when only the downstream Country 3 chooses to be cooperative, in which case the constraints ${E}_{1}-{F}_{1}-{C2}_{3}-{C3}_{3}+{C2}_{7}+{C3}_{7}<0$, ${E}_{2}-{C1}_{3}-{C3}_{6}+{C1}_{7}+{C3}_{8}<0$, and ${E}_{3}-{C1}_{6}-{C2}_{6}+{C1}_{8}+{C2}_{8}>0$ must be satisfied.

**Table 3:** Analysis of equilibrium points by the eigenvalues of the Jacobian matrix *J.*

Condition	Equilibrium point	Sign of eigenvalues	Stability status
1	(0, 0, 0)	(?, ?, ?)	Uncertain stable point
2	(1, 0, 0)	(?, ?, ?)	Uncertain stable point
3	(0, 1, 0)	(?, ?, ?)	Uncertain stable point
4	(0, 0, 1)	(?, ?, ?)	Uncertain stable point
5	(1, 1, 0)	(?, ?, +)	Unstable point
6	(1, 0, 1)	(?, + , ?)	Unstable point
7	(0, 1, 1)	(?, ?, ?)	Uncertain stable point
8	(1, 1, 1)	(?, −, −)	Uncertain stable point

“ + ” means positive sign of eigenvalue; “ − ” means negative sign of eigenvalue; “*?*” means uncertain sign of eigenvalue.

The point (0,1,1) means the two downstream countries choose to cooperate in spite of Country 1 being non-cooperative. For this to occur the following constraints must be met: ${E}_{1}<{F}_{1}$, ${E}_{2}+{C1}_{5}-{C1}_{1}>0$, and ${E}_{3}+{C1}_{4}-{C1}_{2}>0$. The point (1,1,1) signifies cooperation by all countries, which takes place when ${E}_{1}>{F}_{1}$. In this case, the potential benefit of Country 1 due to the cooperation with downstream countries exceeds the benefit that would accrue if it retained all the water for itself. The points (1,1,0) and (1,0,1) cannot be stable solutions because of the positive eigenvalues in their Jacobian matrix. Therefore, there are six probable stable scenarios for the interaction of three countries in the transboundary river basin.

## Numerical examples

**Figure 2:**
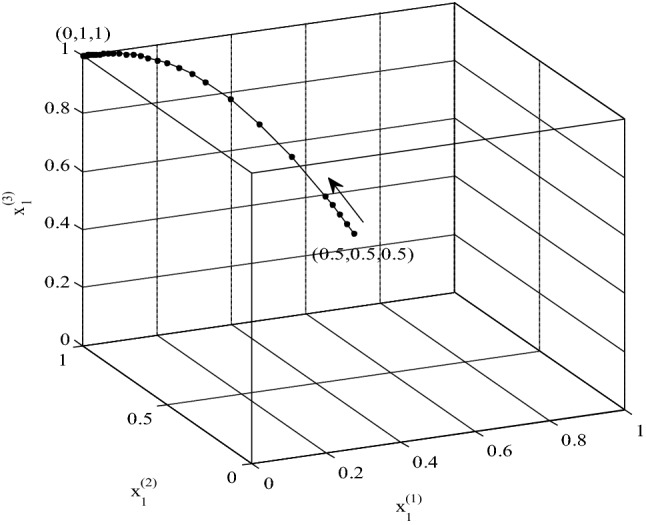
Evolutionary process of the three countries’ strategies (i.e., probabilities of cooperation) with respect to initial values.

The evolutionary tripartite game in the transboundary river basin is illustrated with numerical examples that simulate the evolution of strategies over time. The countries’ decisions and various constraints on potential benefits and conflict costs are examined in an analysis of the evolutionary game. For this purpose, the set of differential equations ${G}^{\left(i\right)}({x}_{1}^{\left(i\right)})$ = 0, for $i=1, 2, 3$, representing evolutionary strategies are solved with the MATLAB software. Each country chooses its strategy at a given time without knowing the strategies of the other countries, and the country repeatedly updates its strategies at subsequent times based on the payoffs of previous time intervals until converging to a stable point of the game. This paper employed hypothetical values of payoff parameters, which is a simplification of real situations. Yet, all is needed in a specific situation is replacing the hypothetical parameters with actual ones and apply this paper methodology thereafter. The assumed values of the benefits and costs are listed in Table 4. Figure 2 shows the ESS point for this choice of benefits and costs is (0,1,1). In this instance, Country 1 has minimum potential benefit and Country 3 has maximum potential benefit. The costs inflicted by country B to others are the largest in this instance, and the costs inflicted by Country 3 to others are the smallest in this instance. Moreover, the water benefit of Country 1 is twice those of Countries 2 and 3. This is so because if Country 1 chooses a cooperative strategy then one-half of the water would have to be shared with Countries 2 and 3.

**Table 4:** Assumed values for the replication dynamics equations’ benefits and costs.

Parameters	Values	Parameters	Values	Parameters	Values	Parameters	Values
*F*_1_	20	*C*_11_	15	*C*_21_	18	*C*_31_	12
*F*_2_	10	*C*_12_	15	*C*_22_	18	*C*_32_	12
*F*_3_	10	*C*_13_	16	*C*_23_	19	*C*_33_	13
*E*_1_	11	*C*_14_	16	*C*_24_	19	*C*_34_	13
*E*_2_	15	*C*_15_	17	*C*_25_	20	*C*_35_	14
*E*_3_	18	*C*_16_	17	*C*_26_	20	*C*_36_	14
		*C*_17_	18	*C*_27_	21	*C*_37_	15
		*C*_18_	18	*C*_28_	21	*C*_38_	15

### Analysis of the evolutionary strategies affected by changing the initial probabilities

**Figure 3:**
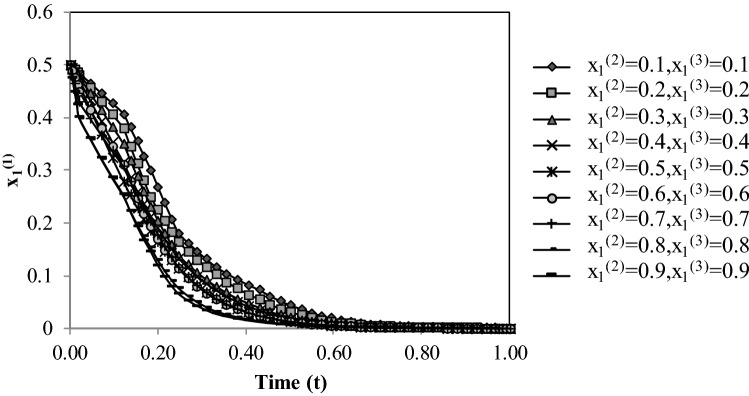
Evolutionary process of probability ${{\varvec{x}}}_{1}^{(1)}$ with respect to initial ${{\varvec{x}}}_{1}^{(2)}$ and ${{\varvec{x}}}_{1}^{(3)}$ values.

The evolution of the countries’ strategies was simulated under the assumed parameters’ values showed in Table 4. Country 1’s initial probability is made equal to ${x}_{1}^{(1)}=$ 0.5, and the effect of changing the initial strategy probabilities of Countries 2 and 3 on the evolution of ${x}_{1}^{(1)}$ is evaluated. It is seen in Fig. 3 that changing the initial values of ${x}_{1}^{(2)}$ and ${x}_{1}^{(3)}$ for Countries 2 and 3, respectively, affects the convergence rate of ${x}_{1}^{(1)}$ to an ESS value. Specifically, as the initial values of ${x}_{1}^{(2)}$ and ${x}_{1}^{(3)}$ increase ${x}_{1}^{(1)}$ converges faster to the final stable value of 0.

**Figure 4:**
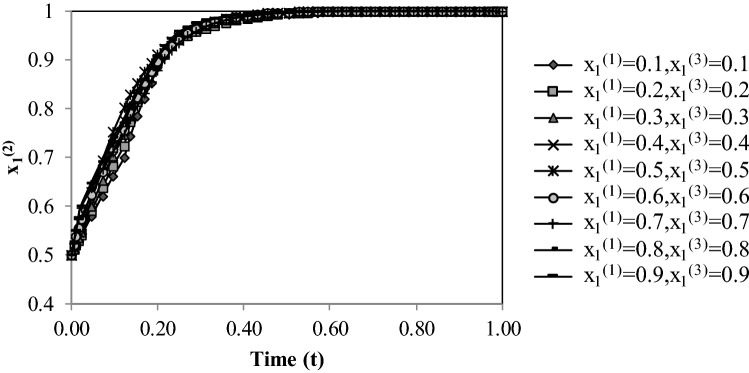
Evolutionary process of probability ${{\varvec{x}}}_{1}^{(2)}$ with respect to initial ${{\varvec{x}}}_{1}^{(1)}$ and ${{\varvec{x}}}_{1}^{(3)}$ values.

Figure 4 displays the evolution of ${x}_{1}^{(2)}$, starting with the initial value of 0.5, by changing the initial values of ${x}_{1}^{(1)}$ and ${x}_{1}^{(3)}$. The initial values of ${x}_{1}^{(1)}$ and ${x}_{1}^{(3)}$ have a negligible effect on the convergence rate of ${x}_{1}^{(2)}$.

**Figure 5:**
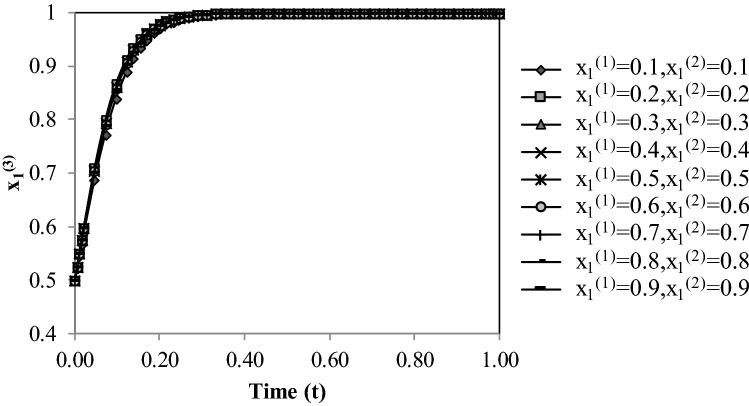
Evolutionary process of probability ${{\varvec{x}}}_{1}^{(3)}$ wit respect to initial ${{\varvec{x}}}_{1}^{(1)}$ and ${{\varvec{x}}}_{1}^{(2)}$ values.

Figure 5 demonstrates the evolution of Country 3’s strategy, starting with the initial value of ${x}_{1}^{(3)}$ = 0.5, corresponding to various initial values of ${x}_{1}^{(1)}$ and ${x}_{1}^{(2)}$. It is evident in Fig. 5 that changing the initial values of ${x}_{1}^{(1)}$ and ${x}_{1}^{(2)}$ does not influence the convergence rate of ${x}_{1}^{(3)}$.

Comparing Figs. 4 and 5 establishes that the probability the probability ${x}_{1}^{(3)}$ converges to a stable point faster than the probability ${x}_{1}^{(2)}$. Country 3 has the most amount of potential benefit, so the probability ${x}_{1}^{(3)}$ reached the value one faster.

Potential benefits would influence the time of convergence of probabilities. For converging the probability ${x}_{1}^{(i)}$ faster to the value of stable point equal to one, the potential benefit of Country *i* should be increased. For converging the probability ${x}_{1}^{(i)}$ faster to the value of stable point which is zero, the potential benefit of Country *i* should be decreased.

### Analysis of the effect of potential benefits on the evolutionary strategies

**Figure 6:**
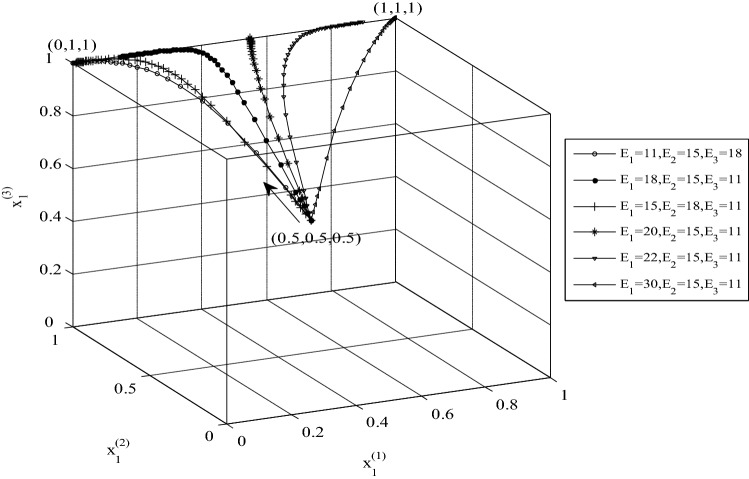
The effect of *E*_*m*_ change on the evolutionary strategies and convergence of strategies to the point (1,1,1).

The simulation of evolutionary strategies was performed to investigate the effect of problem parameters on the interactions of countries in the transboundary river basin. The initial values of the probabilities of the countries’ strategies were set to 0.5, and the evolution of strategies was assessed by changing the countries’ potential benefits. Figure 6 shows the first ESS point of the system is (0,1,1) that evolves towards the final ESS point (1,1,1) by changing *E*_m_. Increasing the values of the potential benefits of the three countries drives convergence towards the ESS point (1,1,1). The ESS point changes from (0,1,1) towards (1,1,1) when the potential benefits of cooperation of Country 1 exceed its water benefit, which clearly is the main condition needed to achieve the full cooperation of countries in the transboundary river basin.

### Analysis of the effect of costs on the evolutionary strategies

**Figure 7:**
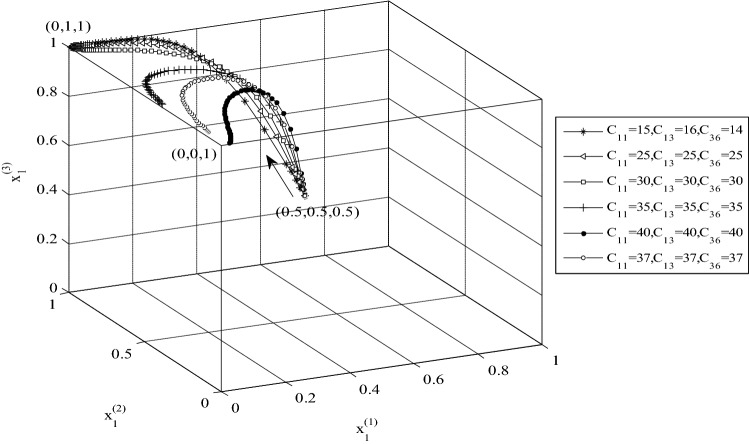
The effect of *C*_11,_ *C*_13_ and *C*_36_ changes on the evolutionary strategies and convergence of strategies to the point (0,0,1).

Two examples of analysis of the evolutionary strategies are presented which involve changing the costs that countries inflict on each by being non-cooperative. The first example’s results are displayed in Fig. 7, which reflects changing *C*_11_ and *C*_13_ (costs of conflict inflicted by Country 1 on Country 2), and *C*_26_ (cost inflicted by Country 3 on 2) and the influence of such changes on the ESS point. Increasing these parameters changes to direction of evolutionary strategies from (0,1,1) towards (0,0,1). The change in the costs satisfies the constraints needed to reach the ESS point (0,0,1) and the evolutionary process reaches this point. In other words, as the costs inflicted by Countries 1 and 3 to Country 2 increase Country 2’s tendency to cooperate decreases and ultimately it chooses the strategy of non-cooperation.

**Figure 8:**
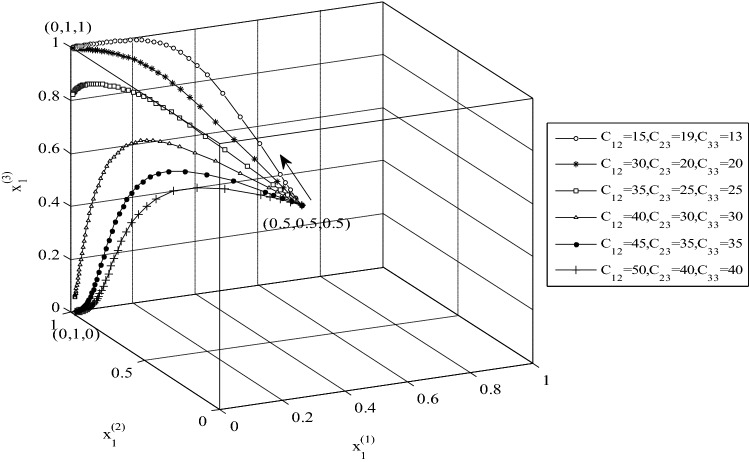
The effect of *C*_11,_ *C*_13_ and *C*_36_ changes on the evolutionary strategies and convergence strategies to the point (0,1,0).

In the second example the values of *C*_12_ (cost inflicted by Country 1 to Country 3), *C*_23_, and *C*_33_ (costs inflicted from Country 2 and 3 on Country 1, respectively) were changed and the effect of the change on the evolutionary strategies is depicted in Fig. 8. The cited cost changes steer the ESS point from (0,1,1,) towards (0,1,0). Increasing the values of the mentioned costs changes the conditions of the system such that the constraints to reach the ESS point (0,1,0) are satisfied.

A noteworthy conclusion emerging from the effects of changing system parameters made is that the convergence of the stable strategies is towards the stable point (0,1,1) when only the parameters of ${a}_{4}$, ${b}_{4}$, ${c}_{4}$ are changed. The presented results demonstrate that the constraints that must be satisfied to reach (0,1,1) have priority, and it is necessary that the constraints ${E}_{2}+{C1}_{5}-{C1}_{1}>0$ and ${E}_{3}+{C1}_{4}-{C1}_{2}>0$ be violated to direct the stable strategies towards (0,0,0), (1,0,0), (0,1,0), and (0,0,1). Also, the constraint associated with realizing ESS (1,1,1) has priority over other constraints, which means when ${E}_{1}>{F}_{1}$ the final stable point is (1,1,1).

## Conclusion

This paper presented an evolutionary method to quantify the long-term strategies of countries located in a transboundary river basin. The evolutionary game theory considers the uncertainty of countries’ strategies. The theory assumes that the strategies change over time in the game which approximates real world situations because the countries located in a transboundary river basin change their strategies and policies from time to time as they interact with each other. Evolutionary game theory was applied to a transboundary river basin in which one country is located upstream (Country 1) and two countries are located downstream (Countries 2 and 3) of the river. All combinations of the countries’ strategies and their payoffs expected from the strategies were defined by a payoff matrix of the tripartite game. Water benefits (i.e. economic benefits of water use) and potential benefits caused by cooperation with other countries (i.e. sustainability benefits) were considered in this paper’s analysis. Conflict costs are defined for the game’s payoffs, which are imposed on countries by their non-cooperative strategies.

The evolutionary stable strategies (ESS) of the problem herein considered were investigated and conditions for reaching these stable strategies were determined. The obtained ESS established the importance of benefits and costs in transboundary river problems. Numerical examples illustrated the evolution of strategies by quantifying the probabilities of cooperation as a function of the initial probabilities of cooperation, benefits, and costs. The decision variables of examples were potential benefits and costs inflicted between countries by non-cooperation. The water benefits to countries were assumed constant in the numerical examples, while potential benefits and conflict costs were variable in assessing the ESSs.

Our assessment of evolutionary strategies and the convergence to stable strategies based on assumed parameters demonstrates that decisions upstream or downstream countries depend on the behavior of the other countries over time. This work paper determined six equilibrium points for the replication dynamics equations, which are the tripartite probability sets (0,0,0), (1,0,0), (0,1,0), (0,0,1), (0,1,1), and (1,1,1) representing the probabilities of cooperation of Countries 1, 2, and 3, respectively.

The results obtained for assumed system parameters indicate the initial strategy probabilities of Countries 2 and 3 effects the evolution of strategy probability of Country 1. Also, the evolutionary strategy of Country 3 converges faster to a stable value equal 1 than Country 2’s, because of its greater potential benefit. Therefore, potential benefits would significantly influence the convergence of strategy probabilities to the stable point. The more potential benefit, the faster convergence to a stable point which is one. Instead, the lower potential benefit, the faster convergence to a stable point of zero.Furthermore, the evolutionary strategies were evaluated by changing the parameters of the replication dynamics equations. The best stable strategy is full cooperation in the basin, i.e., (1,1,1). To reach these strategies the potential benefit to Country 1 by being cooperative must exceed its benefit from water used under a non-cooperative strategy. The application of this paper’s method to transboundary river basin would allow countries to focus on their potential benefits and costs of non-cooperation based on their priorities to achieve expected payoffs from their interactions with other riparian countries in a river basin. As a result, the downstream countries 2 and 3 realize that in order to achieve the cooperation of country 1, it is necessary to increase the amount of potential benefit received by the upstream country.

The role of negotiations in resolving transboundary basin problems can be demonstrated in this article. Countries 2 and 3, for example, can convince Country 1 that cooperation with the downstream countries is worthwhile by granting political and social benefits and even highlighting the value of the environmental benefits of this cooperation.

This paper presented a method for cooperative or non-cooperative strategizing in which one country is upstream and two countries are located downstream in a river basin. The model can be applied to other situations. For instance, when two countries are upstream and one country is downstream. This paper addressed the problem of three riparian countries; yet, the theory is equally applicable to $n\ge 3$ countries, in which case the size of the payoff matrix and the number of probable combinations of strategies grow exponentially.

This paper model involves assumptions worthy of future research. For example, there is one water benefit and one potential benefit for each country in the payoff matrix. Also, there could be incentive benefits or penalty costs imposed by a central governing transboundary agency, even though there are few agencies managing transboundary river basins in most shared river basins nowadays. Future studies could investigate solutions corresponding to benefits and costs arising in specific transboundary water sharing problems. All that would be needed is applying the actual benefits and costs and this paper’ methodology.

## Supplementary Information

Supplementary Information.

## Data Availability

Datasets for this research are included in this paper.
