# Supplementary material for: Analysis of long-term strategies of riparian countries in transboundary river basins
Source: Sci Rep. 2021 Oct 12;11:20199. doi: 10.1038/s41598-021-99655-5 (PMC8511225; doi:10.1038/s41598-021-99655-5)
Supplement: Supplementary file 1 — Supplementary Information. [file 41598_2021_99655_MOESM1_ESM.docx]

# Appendix A

According to the payoff matrix in Table 2 the combinations of the countries’ strategic are as follows:

| $(U_{111}^{\left( 1 \right)},U_{111}^{\left( 2 \right)},U_{111}^{\left( 3 \right)})=(E_{1},F_{2}+E_{2},F_{3}+E_{3})$ | (A1) |
| --- | --- |
| $(U_{112}^{\left( 1 \right)},U_{112}^{\left( 2 \right)},U_{112}^{\left( 3 \right)})=(E_{1}-C_{31},F_{2}+E_{2}-C_{32},F_{3})$ | (A2) |
| $(U_{121}^{\left( 1 \right)},U_{121}^{\left( 2 \right)},U_{121}^{\left( 3 \right)})=(E_{1}-C_{21},F_{2},F_{3}+E_{3}-C_{22})$ | (A3) |
| $(U_{122}^{\left( 1 \right)},U_{122}^{\left( 2 \right)},U_{122}^{\left( 3 \right)})=(E_{1}-C_{23}-C_{33},F_{2}-C_{34},F_{3}-C_{24})$ | (A4) |
| $(U_{211}^{\left( 1 \right)},U_{211}^{\left( 2 \right)},U_{211}^{\left( 3 \right)})=(F_{1},E_{2}-C_{11},E_{3}-C_{12})$ | (A5) |
| $(U_{212}^{\left( 1 \right)},U_{212}^{\left( 2 \right)},U_{212}^{\left( 3 \right)})=(F_{1}-C_{35},E_{2}-C_{13}-C_{36},-C_{14})$ | (A6) |
| $(U_{221}^{\left( 1 \right)},U_{221}^{\left( 2 \right)},U_{221}^{\left( 3 \right)})=(F_{1}-C_{25},-C_{15},E_{3}-C_{16}-C_{26})$ | (A7) |
| $(U_{222}^{\left( 1 \right)},U_{222}^{\left( 2 \right)},U_{222}^{\left( 3 \right)})=(F_{1}-C_{27}-C_{37},-C_{17}-C_{38},-C_{18}-C_{28})$ | (A88) |

The expected payoffs of cooperation ($U_{1}^{(1)})$ and non-cooperation ($U_{2}^{(1)}$) strategies for Country 1, are as follow based on Table 2:

| $U_{1}^{(1)}=x_{1}^{(2)}x_{1}^{(3)}U_{111}^{\left( 1 \right)}+x_{1}^{(2)}x_{2}^{(3)}U_{112}^{\left( 1 \right)}+x_{2}^{(2)}x_{1}^{(3)}U_{121}^{(1)}+x_{2}^{(2)}x_{2}^{(3)}U_{122}^{(1)}$  $=x_{1}^{\left( 2 \right)}x_{1}^{\left( 3 \right)}(E_{1})+x_{1}^{\left( 2 \right)}x_{2}^{\left( 3 \right)}(E_{1}-C_{31})+x_{2}^{\left( 2 \right)}x_{1}^{\left( 3 \right)}(E_{1}-C_{21})+x_{2}^{\left( 2 \right)}x_{2}^{\left( 3 \right)}(E_{1}-C_{23}-C_{33})$  $=x_{1}^{\left( 2 \right)}\left( -C_{31}+C_{23}+C_{33} \right)+x_{1}^{\left( 3 \right)}\left( -C_{21}+C_{23}+C_{33} \right)+x_{1}^{\left( 2 \right)}x_{1}^{\left( 3 \right)}\left( C_{31}+C_{21}-C_{23}-C_{33} \right)+\left( E_{1}-C_{23}-C_{33} \right)$ | (A9) |
| --- | --- |
| $U_{2}^{(1)}=x_{1}^{(2)}x_{1}^{(3)}U_{211}^{\left( 1 \right)}+x_{1}^{(2)}x_{2}^{(3)}U_{212}^{\left( 1 \right)}+x_{2}^{(2)}x_{1}^{(3)}U_{221}^{\left( 1 \right)}+x_{2}^{(2)}x_{2}^{(3)}U_{222}^{\left( 1 \right)}$  $=x_{1}^{\left( 2 \right)}x_{1}^{\left( 3 \right)}\left( F_{1} \right)+x_{1}^{\left( 2 \right)}x_{2}^{\left( 3 \right)}\left( F_{1}-C_{35} \right)+x_{2}^{\left( 2 \right)}x_{1}^{\left( 3 \right)}\left( F_{1}-C_{25} \right)+x_{2}^{\left( 2 \right)}x_{2}^{\left( 3 \right)}\left( F_{1}-C_{27}-C_{37} \right)$  $=x_{1}^{\left( 2 \right)}\left( -C_{35}+C_{27}+C_{37} \right)+x_{1}^{\left( 3 \right)}\left( -C_{25}+C_{27}+C_{37} \right)+x_{1}^{\left( 2 \right)}x_{1}^{\left( 3 \right)}(C_{35}+C_{25}-C_{27}-C_{37})+\left( F_{1}-C_{27}-C_{37} \right)$ | (A10) |

The expected payoff of Country 1 is:

| $U^{(1)}=x_{1}^{(1)}U_{1}^{(1)}+x_{2}^{(1)}U_{2}^{(1)}=\sum_{j=1}^{2} \sum_{k=1}^{2} \sum_{l=1}^{2} x_{j}^{(1)}x_{k}^{(2)}x_{l}^{(3)} U_{jkl}^{(1)}$ | (A11) |
| --- | --- |

$U_{1}^{(2)}$ and $U_{2}^{(2)}$ are expected payoffs of cooperation and non-cooperation strategies for Country 2, respectively. The expected payoffs of the country can be obtained as follows:

| $U_{1}^{(2)}=x_{1}^{(1)}x_{1}^{(3)}U_{111}^{\left( 2 \right)}+x_{1}^{(1)}x_{2}^{(3)}U_{112}^{\left( 2 \right)}+x_{2}^{(1)}x_{1}^{(3)}U_{211}^{(2)}+x_{2}^{(1)}x_{2}^{(3)}U_{212}^{(2)}$  $=x_{1}^{\left( 1 \right)}x_{1}^{\left( 3 \right)}(F_{2}+E_{2})+x_{1}^{\left( 1 \right)}x_{2}^{\left( 3 \right)}(F_{2}+E_{2}-C_{32})+x_{2}^{\left( 1 \right)}x_{1}^{\left( 3 \right)}(E_{2}-C_{11})+x_{2}^{\left( 1 \right)}x_{2}^{\left( 3 \right)}(E_{2}-C_{13}-C_{36})$  $=x_{1}^{\left( 1 \right)}\left( F_{2}-C_{32}+C_{13}+C_{36} \right)+x_{1}^{\left( 3 \right)}\left( -C_{11}+C_{13}+C_{36} \right)+x_{1}^{\left( 1 \right)}x_{1}^{\left( 3 \right)}\left( C_{32}+C_{11}-C_{13}-C_{36} \right)+\left( E_{2}-C_{13}-C_{36} \right)$ | (A12) |
| --- | --- |
| $U_{2}^{(2)}=x_{1}^{(1)}x_{1}^{(3)}U_{121}^{\left( 2 \right)}+x_{1}^{(1)}x_{2}^{(3)}U_{122}^{\left( 2 \right)}+x_{2}^{(1)}x_{1}^{(3)}U_{221}^{(2)}+x_{2}^{(1)}x_{2}^{(3)}U_{222}^{(2)}$  $=x_{1}^{\left( 1 \right)}x_{1}^{\left( 3 \right)}(F_{2})+x_{1}^{\left( 1 \right)}x_{2}^{\left( 3 \right)}(F_{2}-C_{34})+x_{2}^{\left( 1 \right)}x_{1}^{\left( 3 \right)}(-C_{15})+x_{2}^{\left( 1 \right)}x_{2}^{\left( 3 \right)}(-C_{17}-C_{38})$  $=x_{1}^{\left( 1 \right)}\left( F_{2}-C_{34}+C_{17}+C_{38} \right)+x_{1}^{\left( 3 \right)}\left( -C_{15}+C_{17}+C_{18} \right)+x_{1}^{\left( 1 \right)}x_{1}^{\left( 3 \right)}\left( C_{34}+C_{15}-C_{17}-C_{38} \right)+\left( -C_{17}-C_{38} \right)$ | (A13) |

Therefore, the expected payoff of Country 2 is:

| $U^{(2)}=x_{1}^{(2)}U_{1}^{(2)}+x_{2}^{(2)}U_{2}^{(2)}=\sum_{j=1}^{2} \sum_{k=1}^{2} \sum_{l=1}^{2} x_{j}^{(1)}x_{k}^{(2)}x_{l}^{(3)} U_{jkl}^{(2)}$ | (A14) |
| --- | --- |

Respectively, $U_{1}^{(3)}$ and $U_{2}^{(3)}$ represent expected payoffs of Country 3 when chooses cooperative and non-cooperative strategies. According Table 2 the expected payoffs of Country 3 are as follow:

| $U_{1}^{(3)}=x_{1}^{(1)}x_{1}^{(2)}U_{111}^{\left( 3 \right)}+x_{1}^{(1)}x_{2}^{(2)}U_{121}^{\left( 3 \right)}+x_{2}^{(1)}x_{1}^{(2)}U_{211}^{(3)}+x_{2}^{(1)}x_{2}^{(2)}U_{221}^{(3)}$  $=x_{1}^{\left( 1 \right)}x_{1}^{\left( 2 \right)}(F_{3}+E_{3})+x_{1}^{\left( 1 \right)}x_{2}^{\left( 2 \right)}(F_{3}+E_{3}-C_{22})+x_{2}^{\left( 1 \right)}x_{1}^{\left( 2 \right)}(E_{3}-C_{12})+x_{2}^{\left( 1 \right)}x_{2}^{\left( 2 \right)}(E_{3}-C_{16}-C_{26})$  $=x_{1}^{\left( 1 \right)}\left( F_{3}-C_{22}+C_{16}+C_{26} \right)+x_{1}^{\left( 2 \right)}\left( -C_{12}+C_{16}+C_{26} \right)+x_{1}^{\left( 1 \right)}x_{1}^{\left( 2 \right)}\left( C_{22}+C_{12}-C_{16}-C_{26} \right)+\left( E_{3}-C_{16}-C_{26} \right)$ | (A15) |
| --- | --- |
| $U_{2}^{(3)}=x_{1}^{(1)}x_{1}^{(2)}U_{112}^{\left( 3 \right)}+x_{1}^{(1)}x_{2}^{(2)}U_{122}^{\left( 3 \right)}+x_{2}^{(1)}x_{1}^{(2)}U_{212}^{(3)}+x_{2}^{(1)}x_{2}^{(2)}U_{222}^{(3)}$  $=x_{1}^{\left( 1 \right)}x_{1}^{\left( 2 \right)}(F_{3})+x_{1}^{\left( 1 \right)}x_{2}^{\left( 2 \right)}(F_{3}-C_{24})+x_{2}^{\left( 1 \right)}x_{1}^{\left( 2 \right)}(-C_{14})+x_{2}^{\left( 1 \right)}x_{2}^{\left( 2 \right)}(-C_{18}-C_{28})$  $=x_{1}^{\left( 1 \right)}\left( F_{3}-C_{24}+C_{18}+C_{28} \right)+x_{1}^{\left( 2 \right)}\left( -C_{14}+C_{18}+C_{28} \right)+x_{1}^{\left( 1 \right)}x_{1}^{\left( 2 \right)}\left( C_{24}+C_{14}-C_{18}-C_{28} \right)+\left( -C_{18}-C_{28} \right)$ | (A16) |

The expected payoff of Country 3 is:

| $U^{(3)}=x_{1}^{(3)}U_{1}^{(3)}+x_{2}^{(3)}U_{2}^{(3)}=\sum_{j=1}^{2} \sum_{k=1}^{2} \sum_{l=1}^{2} x_{j}^{(1)}x_{k}^{(2)}x_{l}^{(3)} U_{jkl}^{(3)}$ | (A17) |
| --- | --- |

# Appendix B

The replication dynamics equation of Country 1 which are denoted by $G^{(1)}\left( x_{1}^{(1)} \right)$ is as follows:

| $G^{(1)}\left( x_{1}^{(1)} \right)=\frac{dx_{1}^{(1)}}{dt}=x_{1}^{(1)}\left( U_{1}^{(1)}-U^{(1)} \right)=x_{1}^{(1)}x_{2}^{(1)}\left( U_{1}^{(1)}-U_{2}^{(1)} \right)$  $=x_{1}^{\left( 1 \right)}x_{2}^{\left( 1 \right)}\left( x_{1}^{\left( 2 \right)}x_{1}^{\left( 3 \right)}\left( C_{31}+C_{21}-C_{23}-C_{33}-C_{35}-C_{25}+C_{27}+C_{37} \right)+x_{1}^{\left( 2 \right)}\left( -C_{31}+C_{23}+C_{33}+C_{35}-C_{27}-C_{37} \right)+x_{1}^{\left( 3 \right)}\left( -C_{21}+C_{23}+C_{33}+C_{25}-C_{27}-C_{37} \right)+\left( E_{1}-F_{1}-C_{23}-C_{33}+C_{27}+C_{37} \right) \right)$  $=x_{1}^{\left( 1 \right)}x_{2}^{\left( 1 \right)}(a_{1}x_{1}^{\left( 2 \right)}x_{1}^{\left( 3 \right)}+a_{2}x_{1}^{\left( 2 \right)}+a_{3}x_{1}^{\left( 3 \right)}+a_{4})=x_{1}^{\left( 1 \right)}x_{2}^{\left( 1 \right)}A_{0}(x_{1}^{\left( 2 \right)},x_{1}^{\left( 3 \right)})$ | (B1) |
| --- | --- |

where constants $a_{1}$, $a_{2}$, $a_{3}$, $a_{4}$ are as follows:

| $a_{1}=C_{31}+C_{21}-C_{23}-C_{33}-C_{35}-C_{25}+C_{27}+C_{37}$ | (B2) |
| --- | --- |
| $a_{2}=-C_{31}+C_{23}+C_{33}+C_{35}-C_{27}-C_{37}$ | (B3) |
| $a_{3}=-C_{21}+C_{23}+C_{33}+C_{25}-C_{27}-C_{37}$ | (B4) |
| $a_{4}=E_{1}-F_{1}-C_{23}-C_{33}+C_{27}+C_{37}$ | (B5) |

The replication dynamics equation of Country 2 i.e. $G^{(2)}\left( x_{1}^{(2)} \right)$ is:

| $G^{(2)}\left( x_{1}^{(2)} \right)=\frac{dx_{1}^{(2)}}{dt}=x_{1}^{(2)}\left( U_{1}^{(2)}-U^{(2)} \right)=x_{1}^{(2)}x_{2}^{(2)}\left( U_{1}^{(2)}-U_{2}^{(2)} \right)$  $=x_{1}^{\left( 2 \right)}x_{2}^{\left( 2 \right)}\left( x_{1}^{\left( 1 \right)}x_{1}^{\left( 3 \right)}\left( C_{32}+C_{11}-C_{13}-C_{36}-C_{34}-C_{15}+C_{17}+C_{38} \right)+x_{1}^{\left( 1 \right)}\left( -C_{32}+C_{13}+C_{36}+C_{34}-C_{17}-C_{38} \right)+x_{1}^{\left( 3 \right)}\left( -C_{11}+C_{13}+C_{36}+C_{15}-C_{17}-C_{38} \right)+\left( E_{2}-C_{13}-C_{36}+C_{17}+C_{38} \right) \right)$  $=x_{1}^{\left( 2 \right)}x_{2}^{\left( 2 \right)}\left( b_{1}x_{1}^{\left( 1 \right)}x_{1}^{\left( 3 \right)}+b_{2}x_{1}^{\left( 1 \right)}+b_{3}x_{1}^{\left( 3 \right)}+b_{4} \right)=x_{1}^{\left( 2 \right)}x_{2}^{\left( 2 \right)}B_{0}(x_{1}^{\left( 1 \right)},x_{1}^{\left( 3 \right)})$ | (B6) |
| --- | --- |

where constants $b_{1}$, $b_{2}$, $b_{3}$, $b_{4}$ are as follows:

| $b_{1}=C_{32}+C_{11}-C_{13}-C_{36}-C_{34}-C_{15}+C_{17}+C_{38}$ | (B7) |
| --- | --- |
| $b_{2}=-C_{32}+C_{13}+C_{36}+C_{34}-C_{17}-C_{38}$ | (B8) |
| $b_{3}=-C_{11}+C_{13}+C_{36}+C_{15}-C_{17}-C_{38}$ | (B9) |
| $b_{4}=E_{2}-C_{13}-C_{36}+C_{17}+C_{38}$ | (B10) |

The replication dynamics equation of Country 3 denoted by $G^{(3)}\left( x_{1}^{(3)} \right)$ is as follows:

| $G^{(3)}\left( x_{1}^{(3)} \right)=\frac{dx_{1}^{(3)}}{dt}=x_{1}^{(3)}\left( U_{1}^{(3)}-U^{(3)} \right)=x_{1}^{(3)}x_{2}^{(3)}\left( U_{1}^{(3)}-U_{2}^{(3)} \right)$  $=x_{1}^{\left( 3 \right)}x_{2}^{\left( 3 \right)}\left( x_{1}^{\left( 1 \right)}x_{1}^{\left( 2 \right)}\left( C_{22}+C_{12}-C_{16}-C_{26}-C_{24}-C_{14}+C_{18}+C_{28} \right)+x_{1}^{\left( 1 \right)}\left( -C_{22}+C_{16}+C_{26}+C_{24}-C_{18}-C_{28} \right)+x_{1}^{\left( 2 \right)}\left( -C_{12}+C_{16}+C_{26}+C_{14}-C_{18}-C_{28} \right)+\left( E_{3}-C_{16}-C_{26}+C_{18}+C_{28} \right) \right)$  $=x_{1}^{\left( 3 \right)}x_{2}^{\left( 3 \right)}\left( c_{1}x_{1}^{\left( 1 \right)}x_{1}^{\left( 2 \right)}+c_{2}x_{1}^{\left( 1 \right)}+c_{3}x_{1}^{\left( 2 \right)}+c_{4} \right)=x_{1}^{\left( 3 \right)}x_{2}^{\left( 3 \right)}C_{0}(x_{1}^{\left( 1 \right)},x_{1}^{\left( 2 \right)})$ | (B11) |
| --- | --- |

where constants $c_{1}$, $c_{2}$, $c_{3}$, $c_{4}$ are as follows:

| $c_{1}=C_{22}+C_{12}-C_{16}-C_{26}-C_{24}-C_{14}+C_{18}+C_{28}$ | (B12) |
| --- | --- |
| $c_{2}=-C_{22}+C_{16}+C_{26}+C_{24}-C_{18}-C_{28}$ | (B13) |
| $c_{3}=-C_{12}+C_{16}+C_{26}+C_{14}-C_{18}-C_{28}$ | (B14) |
| $c_{4}=E_{3}-C_{16}-C_{26}+C_{18}+C_{28}$ | (B15) |

# Appendix C

The elements of the Jacobian matrix are as follows:

| (C1) | $J_{11}=\left( 1-2x_{1}^{\left( 1 \right)} \right)\left( x_{1}^{\left( 2 \right)}\left( -C_{31}+C_{23}+C_{33}+C_{35}-C_{27}-C_{37} \right)+x_{1}^{\left( 3 \right)}\left( -C_{21}+C_{23}+C_{33}+C_{25}-C_{27}-C_{37} \right)+x_{1}^{\left( 2 \right)}x_{1}^{\left( 3 \right)}\left( C_{31}+C_{21}-C_{23}-C_{33}-C_{35}-C_{25}+C_{27}+C_{37} \right)+\left( E_{1}-F_{1}-C_{23}-C_{33}+C_{27}+C_{37} \right) \right)$ |
| --- | --- |
| (C2) | $J_{12}=x_{1}^{\left( 1 \right)}x_{2}^{\left( 1 \right)}\left( x_{1}^{\left( 3 \right)}\left( C_{31}+C_{21}-C_{23}-C_{33}-C_{35}-C_{25}+C_{27}+C_{37} \right)+\left( -C_{31}+C_{23}+C_{33}+C_{35}-C_{27}-C_{37} \right) \right)$ |
| (C3) | $J_{13}=x_{1}^{\left( 1 \right)}x_{2}^{\left( 1 \right)}\left( x_{1}^{\left( 2 \right)}\left( C_{31}+C_{21}-C_{23}-C_{33}-C_{35}-C_{25}+C_{27}+C_{37} \right)+\left( -C_{21}+C_{23}+C_{33}+C_{25}-C_{27}-C_{37} \right) \right)$ |
| (C4) | $J_{21}=x_{1}^{\left( 2 \right)}x_{2}^{\left( 2 \right)}\left( x_{1}^{\left( 3 \right)}\left( C_{32}+C_{11}-C_{13}-C_{36}-C_{34}-C_{15}+C_{17}+C_{38} \right)+\left( -C_{32}+C_{13}+C_{36}+C_{34}-C_{17}-C_{38} \right) \right)$ |
| (C5) | $J_{22}=\left( 1-2x_{1}^{\left( 2 \right)} \right)\left( x_{1}^{\left( 1 \right)}\left( -C_{32}+C_{13}+C_{36}+C_{34}-C_{17}-C_{38} \right)+x_{1}^{\left( 3 \right)}\left( -C_{11}+C_{13}+C_{36}+C_{15}-C_{17}-C_{38} \right)+x_{1}^{\left( 1 \right)}x_{1}^{\left( 3 \right)}\left( C_{32}+C_{11}-C_{13}-C_{36}-C_{34}-C_{15}+C_{17}+C_{38} \right)+\left( E_{2}-C_{13}-C_{36}+C_{17}+C_{38} \right) \right)$ |
| (C6) | $J_{23}=x_{1}^{\left( 2 \right)}x_{2}^{\left( 2 \right)}\left( x_{1}^{\left( 1 \right)}\left( C_{32}+C_{11}-C_{13}-C_{36}-C_{34}-C_{15}+C_{17}+C_{38} \right)+\left( -C_{11}+C_{13}+C_{36}+C_{15}-C_{17}-C_{38} \right) \right)$ |
| (C7) | $J_{31}=x_{1}^{\left( 3 \right)}x_{2}^{\left( 3 \right)}\left( x_{1}^{\left( 2 \right)}\left( C_{22}+C_{12}-C_{16}-C_{26}-C_{24}-C_{14}+C_{18}+C_{28} \right)+\left( -C_{22}+C_{16}+C_{26}+C_{24}-C_{18}-C_{28} \right) \right)$ |
| (C8) | $J_{32}=x_{1}^{\left( 3 \right)}x_{2}^{\left( 3 \right)}\left( x_{1}^{\left( 1 \right)}\left( C_{22}+C_{12}-C_{16}-C_{26}-C_{24}-C_{14}+C_{18}+C_{28} \right)+\left( -C_{12}+C_{16}+C_{26}+C_{14}-C_{18}-C_{28} \right) \right)$ |
| (C9) | $J_{33}=\left( 1-2x_{1}^{\left( 3 \right)} \right)\left( x_{1}^{\left( 1 \right)}\left( -C_{22}+C_{16}+C_{26}+C_{24}-C_{18}-C_{28} \right)+x_{1}^{\left( 2 \right)}\left( -C_{12}+C_{16}+C_{26}+C_{14}-C_{18}-C_{28} \right)+x_{1}^{\left( 1 \right)}x_{1}^{\left( 2 \right)}\left( C_{22}+C_{12}-C_{16}-C_{26}-C_{24}-C_{14}+C_{18}+C_{28} \right)+\left( E_{3}-C_{16}-C_{26}+C_{18}+C_{28} \right) \right)$ |
